# Comprehensive Review of the Interplay of MicroRNA and Epithelial–Mesenchymal Transition in Radiation Resistance of Cancer

**DOI:** 10.3390/ijms27135781

**Published:** 2026-06-26

**Authors:** Anshu Rajakumar, Qing Cai, Youngman Oh

**Affiliations:** 1 Department of Pathology, Virginia Commonwealth University, Richmond, VA 23284, USA; 2 Massey Comprehensive Cancer Center, Virginia Commonwealth University, Richmond, VA 23284, USA

**Keywords:** radiation therapy, radioresistance, epithelial–mesenchymal transition, microRNA, tumor microenvironment, cancer stem cells, DNA damage response, radiosensitivity

## Abstract

Radiation therapy is a fundamental pillar in cancer treatment, yet its clinical efficacy is frequently compromised by the development of intrinsic and acquired tumor radioresistance. This review provides a comprehensive analysis of the molecular mechanisms underlying radioresistance, with a specific focus on the Epithelial–Mesenchymal Transition (EMT) and its regulation by microRNAs (miRNAs). EMT is recognized as a key driver of therapeutic resistance, enabling cancer cells to acquire enhanced migratory capacity, stem-like characteristics, and resistance to apoptosis. Importantly, ionizing radiation can itself function as a cellular stressor that induces EMT through major signaling pathways, including TGF-β, Wnt, and Notch, thereby establishing a self-reinforcing loop that promotes resistance. In addition, this review highlights the pivotal role of miRNAs as post-transcriptional regulators within this network. Dysregulated miRNAs, acting as either tumor suppressors or oncogenes, modulate EMT-transcription factors and DNA damage repair pathways to influence cellular radiosensitivity. The complex interplay between these factors and the tumor microenvironment is also explored. Finally, emerging therapeutic strategies designed to break this resistance loop, such as EMT inhibitors, miRNA mimics, and antagomirs, as well as combination therapies, are evaluated. Collectively, these approaches hold significant promise for restoring radiosensitivity and improving clinical outcomes in precision oncology.

## 1. Introduction

Radiation therapy (RT) is a fundamental treatment method in the multidisciplinary management of cancer, either as a primary intervention or in conjunction with other therapeutic approaches, such as chemotherapy or surgery [1,2,3]. The therapeutic efficacy of RT stems from its ability to induce cellular damage, primarily by targeting the DNA within malignant cells through two principal mechanisms: direct and indirect effects [4,5]. The direct effect involves the direct transfer of high-energy radiation to DNA molecules, leading to substantial portion of the structural damage induced by radiation. This involves the interaction of radiation energy with surrounding molecules, particularly water, generating highly reactive free radicals that subsequently impede cell growth and division, disrupt cell cycle regulation, and culminate in necrosis, apoptosis, senescence, and autophagy [4]. Despite advancements in RT techniques and precision over recent decades, a substantial proportion of cancer patients continue to face unfavorable prognoses due to the development of tumor radioresistance [5,6]. This resistance manifests either intrinsic or acquired forms, as detailed in Section 2. Distinguishing between these forms is critical, as they arise from different biological drivers ranging from pre-existing genetic mutations to dynamic remodeling of the tumor microenvironment. Consequently, they require different therapeutic interventions [5].

A particularly consequential driver of both metastasis and radioresistance is epithelial–mesenchymal transition (EMT), a highly conserved cellular program fundamental to physiological processes such as embryonic development and wound healing [7,8]. In oncology, the aberrant reactivation of EMT allows polarized, immobile epithelial cells to acquire a motile and invasive mesenchymal phenotype, endowing cancer cells with stem-like properties, survival advantages, and resistance to both chemotherapy and radiation [7,9,10]. The reversibility of this process through Mesenchymal–Epithelial Transition (MET) presents a significant opportunity for therapeutic intervention to restore radiosensitivity [11]. Compounding this complexity, non-coding RNA molecules, specifically microRNAs (miRNAs), have emerged as critical post-transcriptional regulators that govern EMT and broader cancer cell behavior [12,13,14]. By repressing specific target genes, miRNAs modulate cell proliferation and apoptosis; their dysregulation is now recognized as a key driver of therapeutic resistance, making them prime candidates for targeted interventions [15,16].

This review provides a comprehensive overview of radiation therapy resistance in cancer, with a specific focus on the role of miRNA-driven EMT as a central resistance mechanism [5,16]. It first establishes a foundational understanding of radioresistance, then examines EMT’s contribution to cancer progression and treatment failure, followed by a dedicated analysis of miRNA regulatory networks and their interplay with EMT. Finally, it discusses current and emerging therapeutic strategies targeting these mechanisms and concludes with future perspectives and remaining challenges in the field.

## 2. Mechanisms of Tumor Radioresistance

Tumor radioresistance represents a formidable obstacle to cancer eradication, clinically manifesting as a phenomenon where cells either fail to respond to initial therapy or develop resilience over time. This resistance is categorized into two distinct forms. Intrinsic resistance, which exists before radiation exposure due to inherent factors like oncogenic mutations, and acquired resistance, which develops as an adaptive response to the selective pressure of treatment [4,5,6].

At the cellular level, the primary determinant of this resistance is the capacity to manage DNA damage. Since ionizing radiation (IR) exerts its cytotoxic effects primarily by inducing double-strand breaks (DSBs), the efficiency of DNA damage repair (DDR) pathways is critical. Resistant cells often upregulate DDR machinery to resolve these breaks rapidly, indicated by the clearance of γH2AX foci, and dysregulate cell death pathways to evade apoptosis, autophagy, or senescence [4,5,17]. For instance, specific microRNAs like miR-181a have been observed to inhibit radiation-induced apoptosis, directly conferring survival advantages [4,18]. This capacity for repair and survival is not uniform across the tumor; it is frequently concentrated within a subpopulation known as Cancer Stem Cells (CSCs). Characterized by their ability to self-renew and initiate tumor growth, CSCs are inherently more resistant to radiation than bulk tumor cells, possessing superior DNA repair capabilities that drive recurrence [19,20,21,22,23]. Crucially, there is a strong molecular link between these cells and EMT, as the EMT program can induce cancer cells to acquire these robust, stem-like properties [24].

Finally, these cellular mechanisms are supported and amplified by the Tumor Microenvironment (TME). The TME is a complex ecosystem where factors like hypoxia (low oxygen) play a vital role in resistance. Oxygen is essential for the formation of cytotoxic free radicals during irradiation; thus, hypoxic regions naturally limit the effectiveness of radiation damage [25]. Furthermore, the TME undergoes dynamic remodeling during therapy, such as inflammation, which creates a protective niche for tumor cells. Hypoxia also stabilizes Hypoxia-Inducible Factors (HIFs), which activate signaling pathways like TGF-ꞵ and NF-κB to induce EMT, thereby closing the loop between the microenvironment and cellular resistance [5,26,27,28].

## 3. EMT as a Driver of Radioresistance

### 3.1. Molecular and Cellular Hallmarks of EMT

EMT is a dynamic and reversible cellular process characterized by profound morphological and molecular changes, wherein differentiated epithelial cells transform into a mesenchymal phenotype [7,8,29]. This cellular plasticity is essential for various physiological processes, including embryonic development and wound healing, but its aberrant activation is a critical driver of cancer progression and therapy resistance. The transformation during EMT is marked by distinct morphological changes. Epithelial cells typically exhibit a characteristic cobblestone-like appearance, strong cell–cell adhesion, and distinct apical-basal polarity. During EMT, these cells lose their epithelial morphology, detach from neighboring cells, and acquire an elongated, spindle-like fibroblastic shape, along with a redirection of their polarity towards a front-rear axis. At the molecular level, EMT involves comprehensive reprogramming of gene expression:

- Loss of Epithelial Markers: A defining hallmark of EMT is the downregulation or complete loss of key cell–cell adhesion molecules, most notably E-cadherin. This loss of E-cadherin is highly implicated in carcinoma progression, as it allows tumor cells to detach from the primary tumor mass and become more migratory and invasive [30,31]. Concurrently, there is a downregulation of other epithelial proteins such as cytokeratin and claudin.
- Gain of Mesenchymal Markers: Simultaneously, cells undergoing EMT upregulate the expression of mesenchymal proteins, including N-cadherin, Vimentin, and Fibronectin. The shift from E-cadherin to N-cadherin (cadherin switch) is a common feature of EMT, promoting cell motility and invasiveness [30,32].
- Cytoskeletal Rearrangement: The internal actin cytoskeleton undergoes significant reorganization, which facilitates the increased cell motility and invasive capabilities characteristic of mesenchymal cells.

These molecular and morphological changes lead to profound functional consequences. Cellular reprogramming during EMT endows cancer cells with enhanced migratory and invasive capacities, enabling them to disseminate from the primary tumor and metastasize to distant organs. Furthermore, EMT confers resistance to apoptosis, a primary mechanism of cell death induced by many anti-cancer therapies and promotes the acquisition of cancer stem cell-like properties.

The intricate regulatory machinery governing EMT involves a complex network of intracellular signaling pathways and several master transcription factors (EMT-TFs). These EMT-TFs, including members of the Snail family (*SNAI1*, *SNAI2*/Slug), Twist family (*TWIST1*, *TWIST2*), and ZEB family (*ZEB1*, *ZEB2*), along with *PRRX1*, *GOOSECOID*, *E47*, *FOXC2*, *SOX4*, *SOX9*, *HAND1*, and *HAND2*, directly orchestrate the gene expression changes [33,34,35]. They typically bind to E-box sequences in the promoter regions of epithelial genes, repressing their transcription, while also activating the expression of mesenchymal genes. Comprehensive cellular and molecular reprogramming during EMT provides cancer cells with a highly adaptable phenotype that is critical for malignant progression and therapy resistance. The identification of specific EMT-TFs as master regulators of this process offers clear molecular targets for therapeutic intervention aimed at reversing or inhibiting EMT, thereby potentially re-sensitizing cancer cells to therapy.

### 3.2. The Central Role of EMT in Acquiring Radioresistance

EMT is widely recognized as a significant and multifaceted contributor to the acquisition of resistance against various anti-cancer therapies, including radiation therapy. A strong correlation has been observed in experimental settings: inducing EMT in epithelial carcinoma cells often leads to increased radioresistance, and conversely, the induction of radioresistance in cancer cell lines frequently results in a gene expression profile characteristic of EMT [6,19]. This suggests a dynamic and often reciprocal relationship, where EMT not only confers resistance but can also be triggered by the therapeutic stress itself. The mechanisms through which EMT contributes to radioresistance are diverse and interconnected:

- Apoptosis Resistance: Cells that undergo EMT exhibit significantly increased resistance to radiation-induced apoptosis, the primary mechanism by which radiotherapy eliminates tumor cells [36]. Consequently, a subset of tumor cells survives the cytotoxic effects of radiation [37].
- Increased Drug Efflux: A frequently discussed mechanism of drug resistance, which can overlap with radioresistance, is the excessive efflux of therapeutic agents from cells. EMT cells are known to frequently overexpress ATP-binding cassette (ABC) transporters, which are membrane proteins responsible for actively pumping drugs out of the cell. Critically, the promoters of these ABC transporters contain binding sites for EMT-TFs, establishing a direct molecular link between the EMT program and enhanced drug efflux, thereby contributing to multidrug resistance [38,39,40].
- Acquisition of Cancer Stem Cell (CSC) Properties: EMT is a key driver in the acquisition of stem cell properties by cancer cells, leading to the generation of tumor-initiating CSCs [21,22,23,24,41]. These CSCs are inherently more resistant to radiation therapy, possess enhanced DNA repair capabilities, and are a significant cause of tumor recurrence and metastasis following treatment. The close association between EMT and stemness means that EMT can drive radioresistance by promoting the transition of non-CSCs to a CSC-like phenotype.
- Modulation of DNA Damage Repair and Cell Cycle: EMT can influence cellular processes critical for cell survival following radiation exposure, including the efficiency of DNA damage repair and alterations in cell cycle progression. For example, EMT-TFs can affect the expression of genes involved in DNA repair pathways [17].

The bidirectional relationship between EMT and radioresistance, where EMT confers resistance and radiation itself can induce EMT, establishes a self-reinforcing feedback loop. This self-perpetuating cycle is a critical challenge in cancer treatment, as successful therapy can inadvertently select for or induce a more aggressive, resistant phenotype. Consequently, breaking this loop by effectively targeting EMT is considered paramount for overcoming acquired radioresistance and improving long-term therapeutic outcomes for cancer patients.

### 3.3. Radiation-Induced EMT: Molecular Events and Cellular Changes

**Figure 1:**
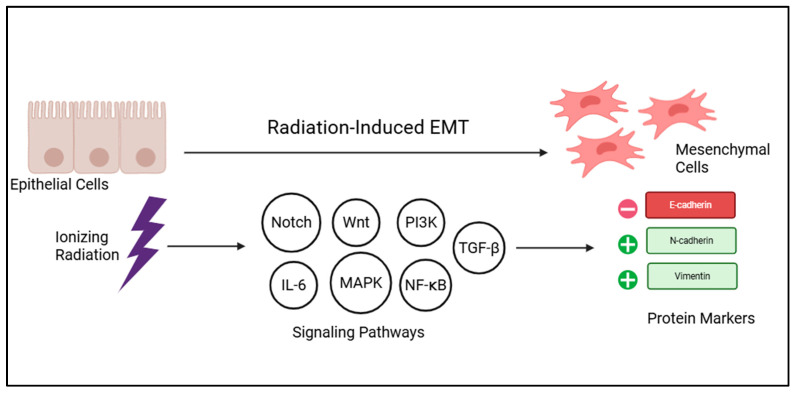
Radiation-Induced EMT and Signaling Pathways. Ionizing radiation activates multiple signaling cascades (Notch, Wnt, PI3K, TGF-β, IL-6, MAPK, NF-κB) that drive the transition of epithelial cells to mesenchymal cells, characterized by loss of E-cadherin and gain of N-cadherin and Vimentin.

As illustrated in Figure 1, IR activates multiple converging signaling cascades. These cascades collectively drive the morphological shift from epithelial to mesenchymal phenotypes, contributing significantly to acquired radioresistance [9,10]. Critically, these pathways do not act independently; rather, they form an interconnected network in which activation of one pathway frequently amplifies another, establishing a self-reinforcing pro-mesenchymal state.

Central to this process is the TGF-ꞵ signaling pathway, a potent inducer of EMT that is readily activated by IR exposure [26,42]. Concurrently, radiation triggers the Wnt/ꞵ-catenin and Notch pathways, which strengthen the expression of master transcription factors like Snail and *ZEB1* [43,44,45]. The cellular stress response to radiation also involves the PI3K/AKT and MAPK/ERK pathways, often activated via the inhibition of suppressors like *PTEN*, which promote cell survival and metastasis [5,26,46,47]. Moreover, the generation of Reactive Oxygen Species (ROS) by IR stimulates inflammatory signaling through NF-κB and IL-6/STAT3, creating a feed-forward loop that sustains the mesenchymal phenotype [5,26,48,49]. These pathways act conjointly to minimize cytotoxicity and reprogram cancer cells into a radioresistant state (see Table 1 for a detailed summary of these pathways and their specific molecular targets).

## 4. The Role of MicroRNAs in Radiation-Induced EMT and Radioresistance

### 4.1. MicroRNA Biogenesis and Mechanisms of Gene Regulation

**Figure 2:**
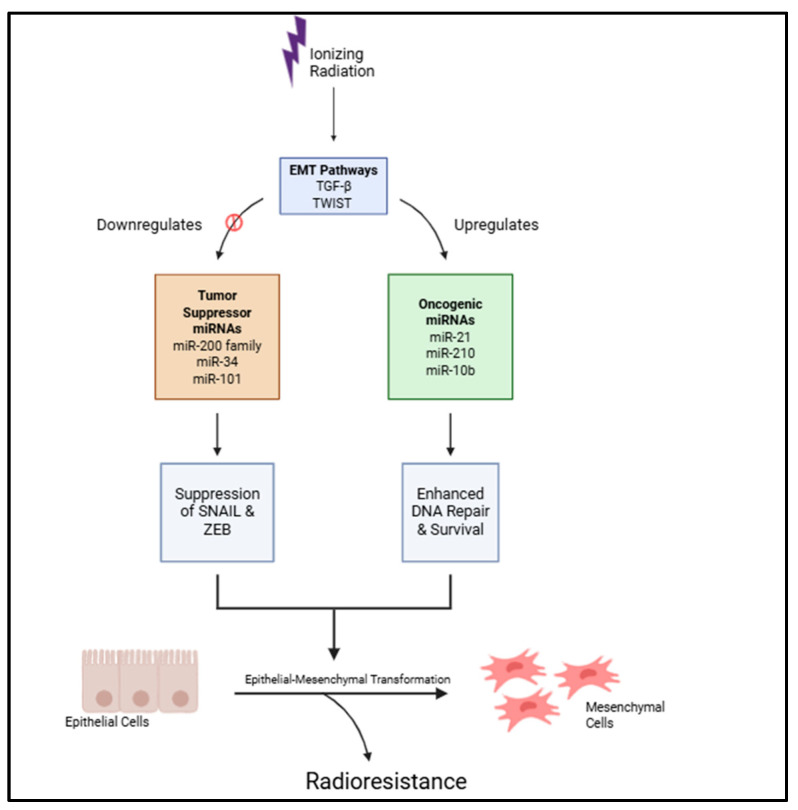
MicroRNA and Mechanisms of Gene Regulation. Ionizing radiation modulates EMT pathways (TGF-β, Twist), which differentially regulate tumor suppressor miRNAs (miR-200 family, miR-34, miR-101) and oncogenic miRNAs (miR-21, miR-210, miR-10b), ultimately influencing epithelial-to-mesenchymal transformation and radioresistance.

MiRNAs are a class of small (approximately 18–22 nucleotides in length) non-coding RNA molecules that exert profound regulatory control over gene expression at the post-transcriptional level [50]. MiRNA biogenesis involves a multi-step process, beginning with transcription into primary miRNA (pri-miRNA) transcripts, which are then processed into precursor miRNA (pre-miRNA) hairpins in the nucleus. These pre-miRNAs are subsequently exported to the cytoplasm, where they are further processed into mature, double-stranded miRNAs. One strand is then incorporated into the RNA-induced silencing complex (RISC). This pathway is visually depicted in Figure 2. The primary mechanism by which miRNAs regulate gene expression involves sequence-specific binding to the 3′-untranslated region (3′-UTR) of target messenger RNA (mRNA) molecules. This binding event, often mediated by a short “seed” sequence (approximately seven nucleotides) within the miRNA, leads to either the degradation of the target mRNA or the inhibition of its translation into protein. This post-transcriptional repression allows a single miRNA to potentially regulate multiple mRNAs simultaneously, making them potent modulators of cellular networks and signaling cascades. MiRNAs are involved in regulating an estimated 30% or more of human genes, with about half of these genes being associated with tumors or located in fragile genomic loci [50]. Their widespread influence means that dysregulation of miRNA expression, whether upregulation or downregulation, can significantly impact various biological and pathological processes, including cancer development, progression, and therapeutic resistance.

### 4.2. MiRNAs Modulating EMT-Related Pathways in Radioresistance

MiRNAs play a critical role in regulating the EMT program, and their dysregulation is frequently implicated in the acquisition of radioresistance by cancer cells. These small non-coding RNAs can either promote or suppress EMT, thereby influencing cellular radiosensitivity through their impact on EMT-related signaling pathways and transcription factors.

One of the most prominent miRNA families in EMT regulation is the miR-200 family (comprising miRs-200a, -200b, -200c, -429, and -141) [12,51]. This family acts as a pivotal suppressor of EMT by directly targeting the mRNA of the key EMT-transcription factors (EMT-TFs) *ZEB1* and *ZEB2* [13,52]. By repressing *ZEB1*/*2*, the miR-200 family helps maintain the epithelial phenotype, characterized by E-cadherin expression, and suppresses the mesenchymal phenotype associated with vimentin. A crucial double-negative feedback loop exists where *ZEB1*/*2* can, in turn, suppress the transcription of miR-200 family members, stabilizing the mesenchymal state and promoting invasion [53]. In the context of radioresistance, upregulation of miR-200b and miR-141 has been shown to reverse TGF-β1-induced EMT and gefitinib resistance in NSCLC cells, suggesting a role in drug resistance [54]. Conversely, miR-200c overexpression has been shown to improve the sensitivity of cancer cells to RT [55].

The miR-34 family (miR-34a, miR-34b, and miR-34c), often regulated by the tumor suppressor p53, directly suppresses EMT by binding to the 3′UTR of *SNAI1* (Snail), a key EMT-TF [50,56,57,58]. Loss of p53 function or mutations can lead to derepression of Snail due to decreased miR-34 levels, promoting EMT. miR-34a, in particular, has been shown to enhance radiosensitivity. Its re-expression can sensitize pancreatic cancer cells to photon irradiation [56,59]. Other notable miRNAs that modulate EMT and influence radioresistance include:

- miR-145: This miRNA has been reported to regulate *ZEB2* expression and can sensitize cancer cells to radiation by targeting an EMT inducer, *PRRX1* [13,60]. Low miR-145 expression is associated with poor responsiveness of rectal cancer patients to neoadjuvant chemoradiation [61].
- miR-124: Similar to miR-145, miR-124 can enhance colorectal cancer cell sensitivity to radiation by inhibiting PRRX1, an EMT regulator and stemness inducer [14,62].
- miR-205: miR-205 exhibits context-dependent activity: in esophageal squamous cell carcinoma, it promotes radioresistance by targeting *PTEN* and activating PI3K/AKT signaling, while in other contexts it suppresses EMT by targeting *ZEB1* and *ZEB2* [26,63].

### 4.3. MiRNAs Directly Influencing Cellular Radiosensitivity

As shown in Figure 2, the regulatory role of miRNAs in the tumor’s radiation response is dynamic, involving intricate interactions between upstream regulators that alter miRNA expression and downstream targets that determine cell survival. Whether a specific miRNA promotes radiosensitivity or confers resistance depends on this network of interactions [5,16,26].

#### 4.3.1. Upstream Regulation by Radiation and Signaling

IR itself can modify miRNA expression profiles. For instance, IR exposure upregulates the oncogenic miR-21 while downregulating members of the lethal-7 family, shifting the cell toward a pro-survival state [5,64]. These expression changes are further modulated by cellular signaling pathways; for example, the tumor suppressor p53 transactivates the miR-200 family, whereas TGF-ꞵ signaling can elevate miR-181 expression [5,16,65,66]. Additionally, epigenetic modifications, such as the methylation of miRNA promoters, can suppress the transcription of radiosensitizing miRNAs like miR-205 [5,67].

#### 4.3.2. Downstream Modulation of DNA Damage Response

A primary mechanism by which miRNAs influence radiosensitivity is the direct regulation of DNA damage response machinery. Several miRNAs target key repair proteins to prevent the resolution of radiation-induced DNA damage.

- miR-101: This miRNA functions as a potent radiosensitizer by directly binding to the 3′-UTR of DNA-PK and ATM mRNA. This reduction in kinase expression impairs both non-homologous end-joining (NHEJ) and homologous recombination (HR), sensitizing tumor cells to radiation [4,14,17,68].
- miR-7: Acting as a tumor suppressor, miR-7 reduces the expression of DNA-PKcs and EGFR. This prolongs the presence of radiation-induced γH2AX foci (a marker of unrepaired DNA), thereby inhibiting efficient repair [5,69].
- miR-18a: Similarly, this miRNA targets ATM, reducing homologous recombination efficiency and increasing sensitivity to IR [5,70].
- Regulation of Cell Cycle, Apoptosis, and Signaling: Beyond DNA repair, miRNAs dictate cell fate by modulating apoptosis and pro-survival signaling pathways.
- Apoptotic Regulators: The impact of miRNAs on apoptosis is often context dependent. For example, miR-181a can sensitize malignant glioma cells by targeting the anti-apoptotic protein Bcl-2 [71]. However, in cervical cancer, its upregulation inhibits radiation-induced apoptosis, conferring resistance [16,18]. Conversely, miR-25 and miR-29 facilitate apoptosis by targeting BIM and MCL1, respectively [5,16,72,73].
- Signaling Pathways: MiRNAs also function as “fine tuners” of major survival cascades. miR-21 reinforces radioresistance by activating the ERK/NF-κB pathway [5,16,74]. In contrast, miR-7 inhibits the PI3K/AKT pathway by targeting upstream receptors like IGFR, *IRS1*, and *IRS2*, effectively cutting off survival signals that would otherwise protect the cell from radiation toxicity [16,75,76].

A comprehensive summary of these miRNAs, their expression patterns in radioresistance, key targets, and associated cancer types is provided in Table 2. 

## 5. Crosstalk Between MicroRNAs and EMT in Radioresistance

### 5.1. Synergistic Mechanisms and Regulatory Loops

EMT and miRNA dysregulation do not operate in isolation in the context of tumor radioresistance. Instead, they form intricate, synergistic mechanisms and complex regulatory loops that collectively contribute to the adaptive survival of cancer cells following radiation therapy [16,26].

**Figure 3:**
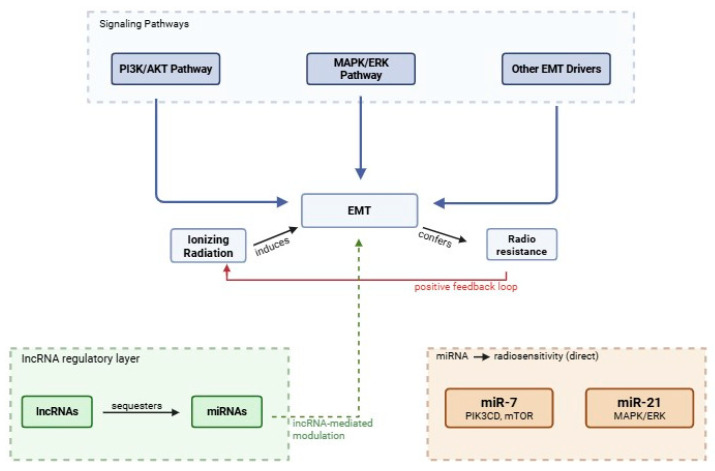
Synergistic Mechanisms and Regulatory Loops Linking miRNA Dysregulation, EMT, and Radioresistance. Key signaling pathways (PI3K/AKT, MAPK/ERK) are fine-tuned by tumor suppressor miRNAs (miR-7) and oncomiRs (miR-21, miR-205), converging on EMT induction and driving radioresistance. Radiation-induced EMT reinforces a self-perpetuating resistance cycle, further modulated by lncRNA-mediated miRNA sponging that amplifies EMT-related gene expression and treatment failure.

A primary level of interaction involves the direct modulation of key signaling pathways that drive EMT by specific miRNAs. As illustrated in Figure 3, the PI3K/AKT pathway, a central mediator of cell survival and EMT, is regulated by numerous miRNAs [46]. MiR-7, a tumor suppressor, acts by targeting *PIK3CD*, mTOR, and p70S6K within this pathway [76]. Similarly, miR-205 can promote radioresistance by inducing EMT through PI3K/AKT signaling [5,26,63]. The MAPK/ERK pathway, another critical pro-survival pathway activated by IR, is also influenced by miRNAs; miR-21, an “oncomiR,” activates this pathway, contributing to radioresistance [5,16]. These examples illustrate how miRNAs can fine-tune the activity of central EMT-driving pathways, thereby indirectly affecting radiosensitivity.

The concept of positive feedback loops is particularly relevant in understanding how these elements reinforce radioresistance. As established in Section 3.2, this radiation-induced EMT creates a self-perpetuating resistance cycle where therapeutic intervention inadvertently promotes a more aggressive and resistant phenotype as visualized in Figure 3 [10,26]. Within this loop, non-coding RNAs (including miRNAs and lncRNAs) are known to regulate EMT and contribute to radioresistance. For instance, lncRNAs can function as molecular sponges for miRNAs, blocking their effects and thereby influencing EMT and drug resistance [16,77,78]. This highlights how the interplay extends beyond direct miRNA-mRNA interactions to include other non-coding RNA species.

The intricate connections between miRNAs and EMT represent a highly dynamic molecular landscape [16]. Understanding these synergistic mechanisms and the precise nature of their regulatory loops is crucial for identifying vulnerable points in the radioresistance network. Disrupting these interconnected pathways, rather than targeting individual components in isolation, holds greater promise for overcoming treatment failure and improving patient outcomes [26].

### 5.2. Complex Interactions Within the Tumor Microenvironment

The TME is not merely a passive bystander but an active participant in shaping the tumor’s response to radiation therapy, profoundly influencing EMT and radioresistance through complex interactions that involve miRNAs [5,26]. The TME is a heterogeneous milieu comprising various cellular components, such as tumor-associated macrophages (TAMs), T cells, neutrophils, NK cells, mast cells, and cancer-associated fibroblasts (CAFs), alongside non-cellular components like the extracellular matrix (ECM), hypoxia, and pH gradients [79,80,81,82].

**Figure 4:**
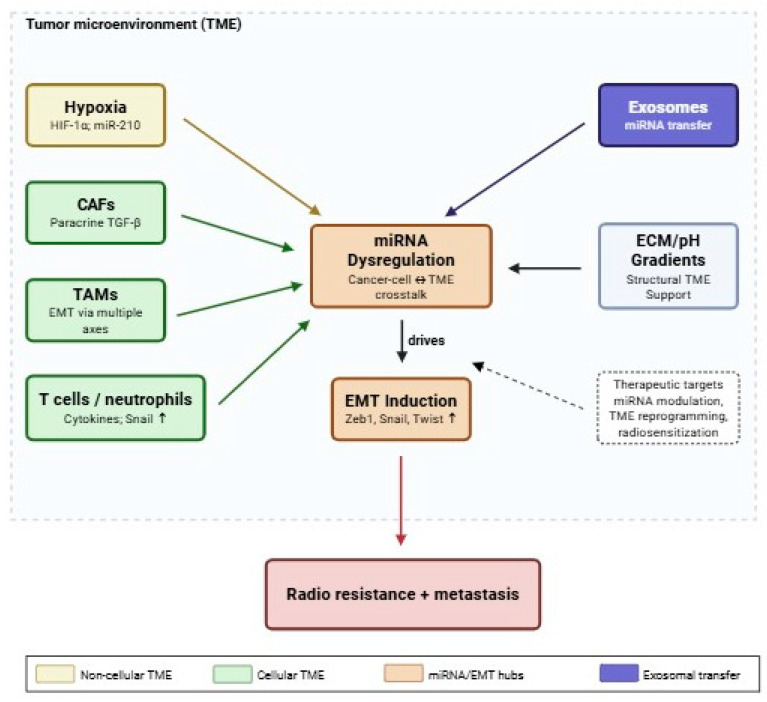
Complex miRNA-Mediated Interactions Within the Tumor Microenvironment Promoting EMT and Radioresistance. Cellular TME components, including CAFs (paracrine TGF-β), TAMs (multi-pathway EMT induction), T cells, and neutrophils (cytokine release, Snail stabilization), alongside non-cellular factors (hypoxia-driven HIF-1α/miR-210, ECM) and exosome miRNA transfer, converge on a central miRNA dysregulation hub that drives EMT transcription factor upregulation (ZEB1, Snail, Twist), ultimately producing a radioresistant, metastatic phenotype.

TME’s Influence on EMT and Radioresistance:

- Hypoxia: A hostile microenvironment, hypoxia, is a significant factor in eliciting EMT. It stabilizes HIFs, which activate key EMT-driving pathways such as TGF-β, NF-κB, and Notch, promoting the expression of EMT-TFs like *ZEB1*, Snail, and Twist. As shown in Figure 4, hypoxia also shares signal pathways with EMT and can confer stem cell-like properties to tumor cells, further contributing to radioresistance [24,25,26,27,28].
- Cellular Components:
  ○ Cancer-Associated Fibroblasts (CAFs): CAFs significantly contribute to EMT by inducing paracrine TGF-β signaling and producing various cytokines and growth factors, including IL-6, EGF, VEGF, and HGF. These factors can promote EMT and foster a radioresistant phenotype [26,83].
  ○ Immune Cells: TAMs induce EMT through multiple signaling pathways (e.g., PI3K/AKT-ERK1/2, COX-2, HIF-1α, EGFR/ERK1/2, Smad/Snail, TGF-β, JAK2/STAT3/miR-506-3p/*FoxQ1* axis) [24,26,84]. Activated T cells can release soluble factors like TNF-α, IL-6, and TGF-β that facilitate EMT-related gene expression [85]. Even neutrophils can exacerbate hypoxia and stabilize Snail, eliciting partial EMT [26].
  ○ Exosomes: Extracellular vesicles, such as exosomes derived from irradiated T cells, can promote metastasis by inducing EMT through increased β-catenin expression and activation of the NF-κB/Snail pathway. Exosomes can also transfer miRNAs, mediating EMT-induced drug resistance. For example, exosomes containing miR-155 from paclitaxel-resistant gastric cancer cells can induce EMT and chemoresistance in sensitive cells [16,26,86,87,88].

Involvement of miRNAs in TME Interactions:

- MiRNAs and TME: MiRNAs are intimately involved in mediating the crosstalk between cancer cells and the TME. For instance, miR-210 is a hypoxia-induced miRNA that is an independent prognostic marker in lung cancer [89]. The dysregulation of miRNAs can transform an unreceptive cancer microenvironment into a cancer-friendly microenvironment, as seen in Figure 4 [16,26].

The complex interactions within the tumor microenvironment, where various cellular and non-cellular components actively induce and maintain EMT and radioresistance, are profoundly influenced by the regulatory actions of miRNAs [5,26]. This intricate network implies that therapeutic strategies must consider the TME as a dynamic entity that can either promote or hinder treatment efficacy. Targeting these microenvironmental interactions, particularly those mediated by miRNAs, represents a promising avenue for overcoming radioresistance [16,26].

## 6. Therapeutic Strategies to Overcome Radioresistance

Overcoming tumor radioresistance is a critical unmet need in oncology. Given the multifaceted nature of radioresistance, particularly its close association with EMT and miRNA, emerging therapeutic strategies are increasingly focusing on targeting these pathways, often in combination with conventional radiation therapy.

### 6.1. Targeting EMT Pathways for Radiosensitization

The reversibility of EMT presents an appealing therapeutic opportunity to re-sensitize cancer cells to radiation. Strategies aimed at inhibiting or reversing EMT include:

- Inhibiting EMT-Related Signaling Pathways: Disrupting the key signaling cascades that drive EMT can effectively block its progression [26].
  ○ Wnt/β-catenin Pathway: Therapeutic agents targeting the Wnt-signaling pathway, such as the porcupine (PORCN) inhibitor LGK-974 (WNT-974), have been evaluated preclinically and in early-phase clinical trials. In preclinical models, LGK-974 inhibits WNT-related gene expression and WNT-dependent phosphorylation of LRP6, demonstrating tumor growth inhibition in pancreatic cancer [90]. However, direct radiosensitization has not yet been established in clinical settings, and Phase I trial data remain preliminary.
  ○ TGF-β Pathway: Inhibitors of the TGF-β pathway represent another strategy for radiosensitization. Preclinically, the TGF-β receptor I kinase inhibitor SB-431542 has been shown to block EMT and cancer stem cell programs, increasing radiosensitivity in breast cancer models [91]. At the clinical level, galunisertib (LY2157299), a small-molecule TGF-βRI inhibitor, has been evaluated in Phase I and II trials across multiple solid tumors, with manageable toxicity reported [92]. Fresolimumab, a pan-TGF-β neutralizing antibody, has been combined with radiotherapy in a Phase I/II study, where it demonstrated the ability to augment radiation-induced abscopal responses in metastatic breast cancer [93]. These clinical results support further investigation of TGF-β inhibition as a radiosensitization strategy, though definitive Phase III evidence is lacking.
  ○ NF-κB Pathway: Blocking NF-κB activity can counteract EMT formation and decrease radioresistance [94]. Denosumab, a monoclonal antibody targeting RANKL, which can activate NF-κB signaling, has been evaluated clinically; however, the ABCSG-18 trial demonstrated that denosumab did not improve disease-free survival outcomes in early breast cancer beyond its established bone-protective effects [95]. Direct radiosensitization through NF-κB inhibition therefore remains an area requiring more targeted clinical investigation.
  ○ PI3K/Akt/mTOR Pathway: Inhibitors of the PI3K/AKT/mTOR axis have shown promise in reducing EMT and CSC markers, though evidence varies considerably by agent and context. Preclinically, the dual PI3K/mTOR inhibitor BEZ235 (dactolisib) decreases EMT marker expression and promotes radiosensitivity in cancer cell lines [96]; however, its clinical development has been substantially limited by toxicity. Simvastatin, a statin repurposed preclinically, has been shown to sensitize radioresistant esophageal cancer cells and reverse EMT via the *PTEN*-PI3K/AKT axis in preclinical models [97], though clinical radiosensitization data remain limited. PF-05212384 and everolimus have been evaluated in clinical trials across multiple solid tumor types, with everolimus demonstrating efficacy in hormone receptor-positive breast cancer [98]; their specific contribution to radiosensitization through EMT modulation remains under investigation.
  ○ Notch Pathway: γ-secretase inhibitors (GSIs) suppress Notch signaling and have been explored as a means of reversing EMT and improving radiosensitivity [43,44]. Among these, RO4929097 has been evaluated in Phase Ib clinical trials, where it demonstrated limited single-agent efficacy, underscoring the need for combination approaches [99]. Preclinically, natural compounds with GSI activity including tangeretin, rhamnetin, and cirsiliol have been shown to suppress Notch signaling and reverse EMT in cancer cell models, though these agents have not yet been evaluated in clinical radiosensitization studies.
  ○ HIF-1-Targeting Agents: Given hypoxia’s role in inducing EMT and radioresistance, agents that suppress HIF-1α represent a rational radiosensitization strategy [28]. Sunitinib and sorafenib, multi-kinase inhibitors that target VEGFR and reduce HIF-1α-driven angiogenesis, have been evaluated preclinically in combination with radiotherapy with evidence of radiosensitization through tumor vasculature normalization and reduction in hypoxic regions [100]. Bortezomib, a proteasome inhibitor that indirectly suppresses HIF-1α, has also demonstrated preclinical radiosensitizing activity. It should be noted that paclitaxel, while used clinically as a concurrent radiosensitizer, acts primarily through mitotic arrest rather than HIF-1α suppression and is more accurately categorized as a cytotoxic radiosensitizer [101]. Clinical evidence for HIF-1α-targeted radiosensitization specifically remains limited, and further trials are needed.
- Targeting EMT-TFs: Direct targeting of master EMT-transcription factors such as Snail, Slug, and Twist represents a promising strategy, as these factors sit at the convergence point of multiple upstream signaling pathways and directly drive the expression of mesenchymal genes [34,35].
- Reversing the EMT Phenotype: Certain compounds can induce MET, thereby re-sensitizing cells. Metformin, an anti-diabetic drug, has demonstrated preclinical activity against EMT and cancer stemness through multiple mechanisms. It has been shown to suppress EMT-transcription factors including *ZEB1*, *TWIST1*, and SLUG in cancer cell models, thereby reducing invasiveness and stem-like properties [102,103]. Preclinical studies have additionally demonstrated radiosensitizing effects of metformin in several cancer types [104]. Clinical evidence for metformin as a radiosensitizer remains limited, and its role in this context is currently under investigation. Berberine can perturb TGF-β-induced EMT and sensitize colon epithelial cancer cells to radiation [105]. The histone deacetylase inhibitor vorinostat has also shown promise in reversing EMT [26,106].

### 6.2. MicroRNA-Based Therapeutic Approaches

Given the critical roles of miRNAs in regulating EMT, cancer stemness, and therapy resistance, miRNA-based therapies represent a promising approach to overcome radioresistance. These strategies typically involve either [5,16,107]:

- MiRNA Mimics: This strategy involves re-introducing tumor-suppressive miRNAs that are downregulated in resistant cells. For example, miR-34a re-expression has been shown preclinically to induce radiosensitization through suppression of Notch-1 signaling in cancer cell models [108]. Clinically, MRX34, a liposomal miR-34a mimic, was advanced into a Phase I trial in patients with advanced solid tumors [109]; however, the trial was terminated early due to severe immune-related adverse events, including five treatment-related deaths, highlighting that immunogenicity and systemic toxicity remain critical unresolved barriers to clinical translation of miRNA mimic therapies. Preclinically, overexpression of miR-100 has been shown to suppress ATM expression in a human glioma cell line (M059J), thereby impairing DNA damage repair and enhancing sensitivity to both chemotherapy and radiation in that model [110]. These findings are currently limited to in vitro evidence and have not been validated in clinical settings.
- Anti-miRNAs (Antagomirs): Inhibiting oncogenic miRNAs that are upregulated in resistant cells. For instance, inhibiting the oncogenic miR-21 using antisense antagomirs has been shown preclinically to enhance apoptosis and reduce radioresistance in glioblastoma cell lines [111]. Clinical translation of anti-miR-21 strategies has not yet been achieved, and evidence remains confined to preclinical models.
- Combination with Conventional Therapies: MiRNA-based therapies are particularly attractive in combination with other anti-cancer therapies due to their ability to target multiple genes associated with resistance-mediating signaling pathways. Curcumin, a natural polyphenol, has been shown preclinically to induce cancer cell death and chemosensitization through modulation of Notch signaling pathways in esophageal cancer models [112]. While curcumin has been reported to influence miRNA expression profiles in some contexts, its extremely poor oral bioavailability represents a significant barrier to clinical translation, and direct evidence of miRNA-mediated EMT suppression as its primary radiosensitizing mechanism remains to be established.

Challenges remain in the clinical translation of miRNA-based therapies, including the development of stable nanoconstructs for delivery, efficient delivery methods to target cells, overcoming speedy excretion, ensuring correct intracellular release, achieving biostability, and addressing potential off-target effects and immunogenicity [16]. However, ongoing research, particularly into nanoparticle or liposome-based microcarriers, offers potential solutions for improving tumor-killing effects and radiosensitivity [113,114].

### 6.3. Emerging Combination Therapies

Given that radioresistance is a multifactorial phenomenon involving numerous interconnected pathways, a single-target approach is often insufficient to achieve durable responses. Therefore, the trend in cancer therapy is increasingly shifting towards combination therapies to overcome resistance mechanisms [5,24].

- Targeted Therapy Combined with Radiotherapy: Combining RT with agents that target specific pro-survival pathways has shown clinical promise. Cetuximab, an anti-EGFR monoclonal antibody, demonstrated significantly improved locoregional control and overall survival when combined with radiotherapy in a landmark Phase III trial in locally advanced head and neck squamous cell carcinoma, establishing it as a clinically validated radiosensitizer [115]. Preclinically, the dual EGFR/HER2 inhibitor lapatinib has also demonstrated radiosensitizing effects through MEK/ERK pathway inhibition and cell cycle arrest, though its clinical radiosensitization data are less mature than those for cetuximab [5]. Similarly, inhibiting the VEGF/VEGFR pathway has been proposed as a means of normalizing tumor vasculature, reducing local hypoxia, and thereby increasing radiosensitivity [116]. Regorafenib, a multi-kinase inhibitor targeting VEGFR among other pathways, has established clinical utility in colorectal and gastrointestinal stromal tumors; however, its specific combination with radiotherapy for radiosensitization purposes has limited clinical evidence and warrants dedicated investigation.
- EMT-Targeting Agents with RT: Combining agents that inhibit or reverse EMT with radiation therapy is a logical strategy to prevent or overcome acquired radioresistance [10,26]. Metformin, an EMT-targeting agent, has been shown to improve progression-free survival when combined with EGFR tyrosine kinase inhibitors in advanced lung adenocarcinoma in a clinical study, suggesting that concurrent EMT suppression may augment the efficacy of targeted therapies [102,117].
- MiRNA-Based Combinatorial Therapy: MiRNAs’ ability to regulate multiple resistance-mediating pathways by targeting multiple genes makes them ideal candidates for combinatorial approaches. Modulating dysregulated miRNA levels (via mimics or inhibitors) can sensitize cancer cells to other anti-cancer therapies, offering a powerful synergistic effect [5,16].
- Targeting CSCs: Strategies that target CSC phenotypes represent another avenue for overcoming radioresistance. The ionophore salinomycin was shown in a foundational preclinical study to selectively eliminate breast cancer stem cells with greater potency than conventional chemotherapy [118]. It has subsequently demonstrated the ability to reduce tumor-initiating cell populations in preclinical models [21,22]. Its potential to enhance radiosensitivity when combined with RT remains largely unexplored clinically and dedicated in vivo and clinical studies are needed.

The development of novel combination strategies is essential to defeating therapeutic resistance. An improved understanding of the cellular basis of cancer therapeutic resistance, particularly the intricate interplay between EMT and miRNAs, provides promising opportunities to design and develop novel, more effective cancer treatment strategies [5,16,24].

## 7. Conclusions and Future Perspectives

### 7.1. Summary of Key Findings and Clinical Implications

Radiation therapy remains a cornerstone of cancer treatment; however, its clinical effectiveness is frequently limited by tumor radioresistance. As discussed throughout this review, radioresistance arises from a complex integration of intrinsic and acquired resistance [5,24,26]. A central theme emerging from this analysis is the pivotal role of EMT. EMT represents a reversible and highly plastic cellular program that endows cancer cells with invasive potential, stem-like features, and critically, resistance to radiation [10,26]. Importantly, the bidirectional EMT–radiation feedback loop described in Section 3.2 contributes to therapeutic failure.

MiRNAs emerge as pivotal regulators within this complex network. These small non-coding RNAs intricately modulate EMT-related pathways and directly influence cellular radiosensitivity by targeting key genes involved in DNA damage response, cell cycle control, and apoptosis [5,16]. Their dysregulation, whether upregulation of oncogenic miRNAs or downregulation of tumor-suppressive ones, profoundly impacts the radioresistant phenotype [16]. The intricate crosstalk between EMT and miRNAs creates a robust resistance mechanism. This interconnectedness underscores that radioresistance is a systemic cellular adaptation rather than an isolated molecular event.

From a clinical perspective, these findings emphasize the need for precision oncology [119]. The dynamic nature of radioresistance necessitates approaches that move beyond conventional single-target therapies. Identifying pre-treatment biomarkers (e.g., specific miRNA profiles) could help predict intrinsic resistance and guide initial treatment choices [5,16]. In addition, understanding the adaptive changes occurring during therapy points towards the development of dynamic, adaptive treatment strategies that can intervene to reverse acquired resistance [5].

### 7.2. Challenges and Future Directions in Research

Despite significant advancements in understanding the molecular underpinnings of radioresistance, several challenges persist in translating this knowledge into effective clinical strategies:

- Complexity of Molecular Mechanisms: The sheer complexity and redundancy of the signaling pathways involved in interactions between EMT and miRNA regulation pose a considerable challenge. Targeting a single pathway may lead to compensatory activation of alternative routes, limiting therapeutic efficacy [5].
- Tumor Heterogeneity and Plasticity: Tumors are highly heterogeneous, containing diverse cell populations, including CSCs and cells in various EMT states. This plasticity allows cancer cells to adapt rapidly to therapeutic pressures, making durable responses difficult to achieve.
- Drug Delivery and Off-Target Effects: For miRNA-based therapies and many targeted agents, developing efficient and safe delivery systems that ensure specific targeting of cancer cells while minimizing off-target effects remains a significant hurdle [16]. Challenges include stable nanoconstructs, appropriate delivery methods, rapid excretion, incorrect intracellular release, poor biostability, endosomal escape, and immunogenicity.

Future research directions should focus on addressing these challenges to pave the way for more effective radiotherapy:

- Integrated Multi-Omics Approaches: Utilizing advanced multi-omics technologies (genomics, transcriptomics, proteomics, metabolomics) to comprehensively map the dynamic changes in EMT and miRNA profiles during and after radiation therapy. This could reveal novel, interconnected targets [5].
- Development of Novel Combination Therapies: Designing rational combination therapies that simultaneously target multiple, synergistic resistance pathways. This includes combining radiation with EMT inhibitors and miRNA mimics/antagomirs. The goal is to achieve synergistic radiosensitization while minimizing toxicity [5,16].
- Advanced Drug Delivery Systems: Investing in the development of sophisticated nanoparticle or liposome-based delivery systems for miRNAs and targeted agents to improve their tumor specificity, bioavailability, and intracellular uptake, thereby enhancing therapeutic index [16,113,114].
- Liquid Biopsies for Dynamic Monitoring: Exploring the utility of circulating miRNAs as non-invasive biomarkers in liquid biopsies to monitor treatment response, detect early signs of acquired resistance, and guide adaptive treatment modifications in real-time [5,16,120].
- Personalized Radiotherapy: Moving towards personalized radiotherapy approaches based on individual patient tumor profiles, including their EMT status and miRNA characteristics, to optimize treatment selection and overcome patient-specific resistance mechanisms [5,119].

In conclusion, overcoming tumor radioresistance remains a critical objective in improving cancer treatment outcomes. The complex interplay between EMT and miRNAs represents both a significant challenge and a promising therapeutic opportunity. Continued rigorous research into these complex molecular networks, coupled with innovative therapeutic design and delivery strategies, holds the promise of transforming the efficacy of radiation therapy in the fight against cancer.

## Figures and Tables

**Table 1:** Key Signaling Pathways Regulating EMT and Radioresistance.

Signaling Pathway	Targeted Molecule/Key Receptor	Function in EMT & Radioresistance
TGF-β Pathway	TGF-β Receptor	Induces EMT, promotes the CSC program, regulates EMT markers
Wnt/β-catenin Pathway	β-catenin, LRP6, Snail	Modulates EMT-related gene expression, increases ALDH activity, and strengthens Snail expression
PI3K/AKT Pathway	PI3K, AKT, *PTEN*	Regulates Snail, Twist, and EMT markers; activated by *PTEN* inhibition
Notch Pathway	Notch Receptors	Promotes expression of *ZEB1*, Slug, Snail, NF-κB, Vimentin; highly activated by IR
NF-κB Pathway	NF-κB, IκB	Regulates Twist, Snail, SIP1; involved in EMT-mediated radioresistance; activated by IR
IL-6/STAT3 Pathway	*IL-6R*, STAT3	Regulates expression of *ZEB1* and mesenchymal markers; mediates progression and resistance
MAPK/ERK Pathway	MEK, ERK	Promotes cell survival, proliferation, and differentiation; upregulates Snail; protects from IR cytotoxicity

**Table 2:** Selected MicroRNAs Involved in EMT and Radiosensitivity/Radioresistance.

miRNA	Expression in Radioresistance	Key Target Genes/Pathways	Functional Impact on EMT & Radiosensitivity	Reported Cancer Type(s)
miR-200 family	Downregulated in EMT/Resistance	*ZEB1*, *ZEB2*	Suppresses EMT; Enhances radiosensitivity	NSCLC; Lung cancer
miR-34 family	Downregulated in EMT/Resistance	*SNAI1* (Snail), *IL-6R*, *ZNF281*	Suppresses EMT; Enhances radiosensitivity; Reverses resistance-induced EMT/stemness	Pancreatic cancer; General p53-dependent contexts
miR-21	Upregulated in EMT/Resistance	*PTEN*, EGFR/STAT3, *HBP1*, ERK/NF-κB	Promotes radioresistance; Induces tumor angiogenesis & metastasis; Reinforces invasiveness	Esophageal squamous cell carcinoma; Glioblastoma
miR-145	Low expression in radioresistance	*ZEB2*, *PRRX1*, Snail	Suppresses EMT; Enhances radiosensitivity; Associated with poor response to chemoradiation	Rectal cancer; Colorectal cancer
miR-124	Enhances sensitivity	*PRRX1*	Enhances radiosensitivity by inhibiting the EMT regulator and stemness inducer	Colorectal cancer
miR-205	Context-dependent (promotes/suppresses)	PI3K/AKT, *ZEB1*, *ZEB2*, SRC	Promotes radioresistance (via PI3K/AKT); Suppresses EMT (via *ZEB1*/*2*); Facilitates invasion (via *PTEN* inhibition)	Esophageal squamous cell carcinoma
miR-7	Tumor suppressor	EGFR, IGFR, *IRS1*, *IRS2*, *PIK3CD*, mTOR, p70S6K	Increases radiosensitivity; Regulates tumor cell survival and proliferation	Glioblastoma
miR-101	Tumor suppressor	DNA-PK, ATM, *ZEB1*	Sensitizes tumor cells to radiation by influencing DDR; Inhibits TGF-β1-induced EMT	General tumor cell models
miR-181a	Context-dependent (sensitizes/confers resistance)	Bcl-2, PRKCD, ATM	Sensitizes glioma cells (via Bcl-2); Confers radioresistance in cervical cancer (via PRKCD); Regulates CSC properties (via ATM)	Malignant glioma; Cervical cancer
miR-10b	Promotes EMT	Twist, *RBICC1*, *PTEN*/PI3K/AKT	Promotes CSC features and invasiveness; Regulated by TWIST and TGF-β	General/breast cancer contexts
miR-210	Upregulated by hypoxia	Snail	Promotes EMT in hypoxic TME	Lung cancer

## Data Availability

No new data were created or analyzed in this study. Data sharing does not apply to this article.
